# Recovery of undamaged electron-density maps in the presence of damage-induced partial coherence in single-particle imaging

**DOI:** 10.1107/S2052252520013019

**Published:** 2020-10-20

**Authors:** Alexander Kozlov, Timur E. Gureyev, David M. Paganin, Andrew V. Martin, Carl Caleman, Harry M. Quiney

**Affiliations:** a ARC Centre of Excellence in Advanced Molecular Imaging, School of Physics, University of Melbourne, Parkville, Victoria 3010, Australia; b Faculty of Health Sciences, University of Sydney, Sydney, NSW 2006, Australia; c School of Science and Technology, University of New England, Armidale, NSW 2351, Australia; d School of Physics and Astronomy, Monash University, Clayton, Victoria 3800, Australia; e School of Physics, RMIT University, Melbourne, Victoria 3000, Australia; f Department of Physics and Astronomy, Uppsala University, PO Box 516, SE-751 20 Uppsala, Sweden; g Center for Free-Electron Laser Science, DESY, Notkestraße 85, DE-22607 Hamburg, Germany

**Keywords:** radiation damage, XFELs, partial coherence, single-particle imaging

## Abstract

Resolving the electronic structure of single biological molecules in their native state was among the primary motivations behind X-ray free-electron lasers. The ultra-short pulses they produce can outrun the atomic motion induced by radiation damage, but the electronic structure of the sample is still significantly modified from its original state. This paper explores the decoherence of the scattered signal induced by temporal evolution of the electronic structure in the sample molecule. It is shown that the undamaged electron density of a single-molecule sample can often be retrieved using only the two most occupied modes from the coherent mode decomposition of the partially coherent diffraction fluence.

## Abstract 2

Single-particle imaging using X-ray free-electron lasers requires an intensive X-ray fluence which damages the sample as it is imaged. This paper explores the potential for including theoretical simulations of single-particle electrodynamics into a reconstruction of the electron density imaged using X-ray free-electron lasers. While partial coherence, induced by radiation damage, poses a challenge for phase retrieval, it is demonstrated that additional coherent modes enable suppression of the effect of radiation damage in the reconstructed sample electron density.

## 1. Introduction   

Single-particle imaging (SPI) using X-ray free-electron lasers (XFELs) seeks to achieve atomic resolution (∼1 Å) in a reconstructed three-dimensional (3D) electron density of a non-crystalline sample, such as single biological molecules, obtained from a large number of two-dimensional (2D) X-ray diffraction patterns (Sayre *et al.*, 1998 ▸; Chapman *et al.*, 2006 ▸; Aquila *et al.*, 2015 ▸). These 2D diffraction patterns are collected in the far field (Fresnel number much smaller than unity) from randomly oriented copies of the sample, each of which is destroyed by the ultra-high X-ray flux (Neutze *et al.*, 2000 ▸). Elastic scattering in light elements of biological importance at X-ray photon energies 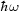 ≃ 10 keV, needed to achieve atomic resolution, is relatively weak. In fact inelastic interactions such as photoionization dominate over the Thompson scattering at these X-ray energies. Photoionization starts a cascade of secondary ionization processes which together turn the sample into a highly charged molecular ion that expands rapidly due to Coulomb repulsion (Hau-Riege *et al.*, 2004 ▸; Fortmann-Grote *et al.*, 2017 ▸).

While radiation damage is a recognized limitation for SPI (Aquila *et al.*, 2015 ▸), a range of technical challenges currently constrain the achievable resolution (Chapman, 2019 ▸). These challenges include sample delivery, background scattering by a carrier medium, sample reproducibility and purity, low number of photons scattered by individual samples, and various sources of detection noise. For a more detailed discussion we refer the reader to Chapman (2019 ▸) and references therein. In this work we focus on the following question, raised by Aquila *et al.* (2015 ▸): ‘Will it be possible to recover undamaged electron-density maps based on damaged sample data?’.

Alongside atomic motion, radiation damage induces electronic structure change in the sample during the pulse (Hau-Riege *et al.*, 2007*a* ▸; Son *et al.*, 2011 ▸). The changes in electron structure, caused by photoionization (Neutze *et al.*, 2000 ▸) and a cascade of secondary ionization events during the pulse, occur on a sub-femtosecond timescale (Seibert *et al.*, 2010 ▸). In nanocrystalline samples the scattering from the damaged structure is suppressed by a breakdown in crystalline period­icity and a loss of bound electrons (Barty *et al.*, 2012 ▸; Caleman *et al.*, 2015*b* ▸; Nass *et al.*, 2020 ▸). In single-molecule samples a reduction in the contrast of speckles in continuous diffraction fluence is caused by the damage-induced thermal motion of atoms (Martin *et al.*, 2015 ▸). If a molecule is confined to a buffer, for example a droplet, the atomic motion will be suppressed compared with a molecule in a vacuum (Hau-Riege *et al.*, 2007*b* ▸). Temporal variation of the electronic structure leads to a loss of coherence in the scattered X-ray photons (Quiney & Nugent, 2011 ▸; Gorobtsov *et al.*, 2015 ▸; Martin & Quiney, 2016 ▸; Caleman *et al.*, 2020 ▸) and significantly modifies the atomic structure factors (Hau-Riege *et al.*, 2007*a* ▸; Son *et al.*, 2011 ▸). This partial coherence can be described in terms of coherent modes (Mandel & Wolf, 1995 ▸, Quiney & Nugent, 2011 ▸), so that the partially coherent scattered intensity is represented as the sum of two or more coherent modes with a certain number of photons occupying each of them. Hence, electron-density maps recovered from a single coherent mode depict a damaged electron structure. Even if atomic motion is insignificant, the depicted electron structure is modified from its initial state. Below we use the term ‘damaged electron density’ in this sense. This damaged electron density tends towards a pulse-averaged distribution in the limit of low incident fluence (Martin & Quiney, 2016 ▸). An application of phase retrieval algorithms which assume full coherence and retrieve the phase of a single mode will therefore yield such a damaged electron density. Generalized phase retrieval algorithms capable of retrieving multiple coherent modes have been reported in the literature (Whitehead *et al.*, 2009 ▸; Quiney, 2010 ▸; Sala *et al.*, 2019 ▸).

In this paper we investigate the possibility of utilizing a sample electrodynamics theory combined with experimental measurements of partially coherent diffraction data to overcome the damage-induced electronic structure changes in the retrieved electron density of the sample. We demonstrate that a linear combination of retrieved coherent modes with modal weights obtained from the theory yields a reconstruction of local atomic electron densities with significantly suppressed radiation damage effects. This reconstruction requires input from a theoretical ionization-dynamics model which relies only on knowledge of the chemical composition of the sample without any structural information. Addition of a second mode significantly improves the reconstruction of undamaged electron density compared with that obtained with a single coherent mode, conventionally used under an assumption of full coherence in the scattered X-ray intensity. The coherence of the scattered signal as a function of the pulse length and fluence is investigated for a model system of DNA origami. These artificial structures have recently been proposed for testing SPI using XFELs (Xavier & Chandrasekaran, 2018 ▸) and cryo-electron microscopy (Kopatz *et al.*, 2019 ▸). For typical contemporary XFEL pulse parameters, as many as 8% of photons can occupy modes other than the primary one. For the theory described below these photons serve as a source of useful structural information rather than decoherence, as would be the case for a conventional approach with an assumption of full coherence. We further discuss the inclusion of additional modes, beyond the first two, into the reconstruction of undamaged electron density.

## 2. Modal decomposition   

In the context of SPI we consider the problem of reconstruction of the 3D electron-density distribution in a sample from the 3D intensity distribution of scattered X-rays. The latter is obtained by merging many 2D diffraction patterns after solving for sample orientations in each pattern. We assume that the incident X-ray pulse is a quasi-monochromatic plane wave propagating along the optic axis *z* with intensity distribution *I*(*x*, *y*, 0, *t*) = *I*_in_(*t*), which is spatially uniform in the object plane *z* = 0 but can vary in time and has a finite duration. Here, (*x*, *y*) denote coordinates in planes perpendicular to the optic axis, and *t* denotes time. The phase distribution of the incident pulse, ϕ(*x*, *y*, 0, *t*) ≃ exp(−*i*ω*t*), is assumed to be approximately flat over the pulse duration *T* within the area occupied by the sample in the object plane, where ω = 2π*c*/λ, *c* is the speed of light in a vacuum and λ is the mean wavelength. We also assume that the scattering is weak, so that the standard first Born approximation can be applied to describe the diffracted intensity in a remote detector plane *z* = *R*. Under these assumptions, the integrated intensity of diffracted X-rays *W*(**q**) in the far (Fraunhofer) field, *R* ≫ *d* ^2^/λ, where *d* is the diameter of the sample, obtained by integrating the diffracted intensity *I*_*R*_(**q**, *t*) over a single exposure (pulse length) time, can be expressed as 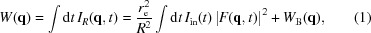


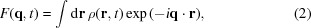
where the molecular form factor *F*(**q**, *t*) is the Fourier transform of the time-dependent molecular electron density ρ(**r**, *t*), *r*_e_ is the classical electron radius, and *W*_B_(**q**) is the background integrated intensity that contains both the Compton scattering term and other incoherent contributions (Lorenz *et al.*, 2012 ▸; Slowik *et al.*, 2014 ▸; Gorobtsov *et al.*, 2015 ▸). Below we shall assume that background subtraction has been carried out and set *W*_B_(**q**) = 0. In contrast with previous publications (Lunin *et al.*, 2015 ▸; Gureyev *et al.*, 2018 ▸), here we include plasma effects and consider entire atomic configurations (Son *et al.*, 2011 ▸) rather than individual atomic orbitals (Quiney, 2010 ▸; Lunin *et al.*, 2015 ▸) in the damage model during illumination by the incident X-ray pulse. Note that because of the stochastic character of radiation damage processes and the variability of the repeated illumination pulses, the right-hand side of equation (1) is a stochastic distribution; this is taken into account below.

We adopt an independent-atom model for the total mol­ecular density (Quiney & Nugent, 2011 ▸; Son *et al.*, 2011 ▸), so that the total molecular electron density ρ(**r**, *t*) is given by the sum of the atomic electron densities 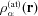 of all atoms in the molecule, 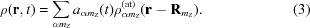
Here α = {*Z*, γ}, and 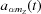 represents the occupancy of the electronic configuration γ in the *m*th atom of chemical element *Z*, located at a position 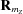 in the molecule. For example, γ = 1 in carbon enumerates the configuration 1*s* ^2^2*s* ^2^2*p* ^2^, γ = 2 stands for 1*s* ^2^2*s* ^2^2*p* ^1^, and so on for all 27 configurations. This treatment of 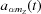 as entire configuration occupancies is in contrast to the original model (Quiney, 2010 ▸), which treated these quantities as the number of electrons in an orbital γ of atom *Z*. This allows us to account for the change in atomic orbitals between different configurations, which can be significant (Son *et al.*, 2011 ▸), especially for heavier elements (Kozlov & Quiney, 2019 ▸).

Substituting the electron density (3) into (1), we can write the intensity integrated over the X-ray pulse duration as
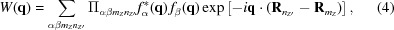


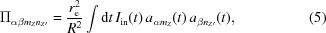
where 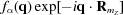 is the Fourier transform of the atomic electron density 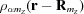, so that *f*_α_(**q**) is an atomic form factor. Here the temporal pulse variability and radiation damage effects are contained in the stochastic ‘damage matrix’ 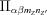. Note that the quantities 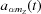 themselves depend on the incident pulse intensity *I*_in_(*t*), as the time-dependent intensity drives the electron-density dynamics of atoms in the molecule. The XFEL pulse is assumed to be sufficiently short that the atomic positions 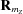 of atoms do not change during the pulse.

The diffracted intensity in equation (4) is a statistical function of repeated measurements. The mean value over the ensemble is equal to 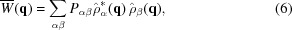
where 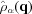 = *f*_α_(**q**)*T*_*Z*_(**q**) and the structure function *T_Z_*(**q**) = 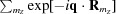 has been introduced. Note that 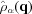 is the Fourier transform of the electron density of all atoms *Z* in the sample, each of which has an electronic configuration denoted by γ. Within the framework of the independent-atom model, the mean damage is independent of the position of each atom in the sample (Hau-Riege *et al.*, 2004 ▸; Lorenz *et al.*, 2012 ▸), so that 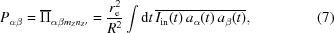
where the atomic position in *a*_α_(*t*) has been dropped, and the overline denotes the average value over an ensemble of damage scenarios. The independent-atom model assumes that, on average, each atom of chemical element *Z*, illuminated by incident X-rays, has the same charge density 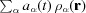 at any time *t*.

The average integrated intensity (6) can be rewritten in diagonal form, 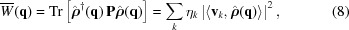
where the vector 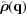 has components 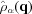, and **v**_*k*_ is an eigenvector of the matrix **PS**, so that **PSv**_*k*_ = η_*k*_ **v**_*k*_, and (**S**)_αβ_ = 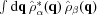 is the overlap matrix (see Appendix *A* [App appa] for details). In the above equation the eigenvalues η_*k*_ are associated with the occupancies of coherent modes,
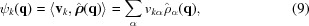
which are orthonormal, so that equation (8) takes the form of a modal decomposition (Mandel & Wolf, 1995 ▸; Quiney, 2010 ▸; Quiney & Nugent, 2011 ▸; Lorenz *et al.*, 2012 ▸; Lunin *et al.*, 2015 ▸) that reflects the coherence properties of the mean integrated intensity 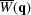. The mode occupancy η_*k*_ is proportional to the total number of photons 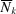 in the mode ψ_*k*_(**q**). Indeed, the mode occupancy can be written as η_*k*_ = 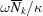 (in atomic units, used henceforth in this article, the reduced Planck constant 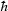 is set to unity and ω = 2πν), where κ is the quantum efficiency of the detector (Goodman, 1985 ▸; Mandel & Wolf, 1995 ▸; Gureyev *et al.*, 2017 ▸).

Now consider the problem of extracting the sample electron density from the coherent modes. This can be achieved using a set of vectors **u**_*k*_ = **Sv**_*k*_ which are bi-orthogonal to vectors **v**_*k*_: 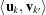 = 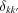. The vectors **u**_*k*_ are eigenvectors of the matrix **SP** so that 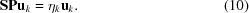
Expanding the vector 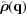 we obtain 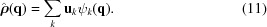
The undamaged electron density can be expressed as ρ_1_(**r**) = 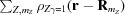, where the index γ = 1 indicates the initial undamaged configuration of each atom. The Fourier transform of the undamaged electron density 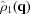 = 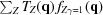 = 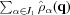, where *J*_1_ = {*Z*, γ = 1} is a subset of all electronic configurations that includes only those that correspond to undamaged atoms. Therefore, 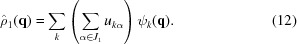



Equation (12) expresses the undamaged electron density in the sample via the coherent modes that can be retrieved from experimental data, collected in the presence of radiation damage, using phase retrieval algorithms (Whitehead *et al.*, 2009 ▸; Quiney, 2010 ▸; Sala *et al.*, 2019 ▸) and the eigenvectors of the matrix **SP** obtained from the theoretical model. Importantly, the first summation in equation (12) can always be truncated at a finite *k* = *M*, which corresponds to the singular-value decomposition (SVD) of the matrix **SP**, **SPx** = 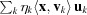 (Golub & Van Loan, 1989 ▸). However, if the sum in equation (12) is truncated so that only *M* < *N* modes are included, where *N* is the total number of modes, then 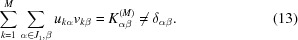
This leads to imperfect reconstruction of the undamaged electron densities using equation (12). We introduce the normalization factor Ω^(*M*)^ = 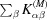 ≠ 1 for *M* < *N*, which rapidly converges to unity when several modes with the highest eigenvalues η_*k*_ are included in the summation. For *M* = 2 (while *N* ≃ 10^3^), Ω^(*M*)^ differs from unity by no more than 40% for all the simulation parameters in Table 1 ▸ that we considered. To correct the reconstructed electron density, we introduce Ω^(*M*)^ into equation (12) and obtain 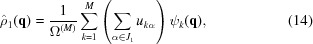
for the norm-adjusted undamaged electron-density reconstruction from the truncated sum over coherent modes. If the radiation damage is negligible, the integrated intensity 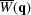 is fully coherent and equation (14) becomes exact with just a single mode occupied by all scattered photons.

In summary, equation (14) demonstrates that the electronic structure corresponding to a sample that is unaffected by radiation damage can potentially be retrieved from only the first two coherent modes ψ_*k*_(**q**) and their occupancies η_*k*_. All coefficients in equation (14) depend only on the chemical composition of the sample and can be obtained via a radiation damage simulation, while the coherent modes ψ_*k*_(**q**) contain information about the sample structure. These modes can in principle be retrieved from the 3D diffraction fluence, collected in the presence of radiation damage, and *a priori* information about the sample (Quiney, 2010 ▸; Quiney & Nugent, 2011 ▸; Curwood *et al.*, 2013 ▸). A different algorithm for solving a generalized phase retrieval problem (Thibault & Menzel, 2013 ▸) has been successfully used to retrieve ten coherent modes from the diffraction data collected in an XFEL imaging experiment (Sala *et al.*, 2019 ▸).

## 3. Radiation-damage dynamics model   

Finding the undamaged electron density (14) from the coherent modes ψ_*k*_(**q**) given by equation (14) requires the expansion coefficients *u*_*k*α_ given by equation (10). The latter form eigenvectors **u**_*k*_ of the matrix **SP**, so finding them requires knowledge of both the overlap matrix (**S**)_αβ_ = 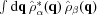 and the damage matrix **P**. According to equation (7), the damage matrix depends on the electron-density occupancies *a*_α_(*t*), which are determined by the ionization dynamics of the sample. This section describes finding these occupancies and modelling the ionization dynamics of the molecule imaged by an XFEL.

In the independent-atom model that was adopted in the derivation of the coherent mode expansion (6), the molecular electron density is approximated by those of individual atoms. Adopting the average-over-configuration description of electron configurations in atoms (Lindgren & Morrison, 1986 ▸; Son *et al.*, 2011 ▸; Kozlov & Quiney, 2019 ▸) and using Parseval’s theorem, the overlap matrix can be written as 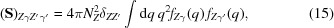
where *N*_*Z*_ is the number of atoms of element *Z*. The atomic scattering form factor *f*_*Z*γ_(*q*), introduced in equation (4), depends only on the absolute value of **q** for a spherically symmetric atomic electron density. The overlap matrix (15) depends only on the atomic scattering form factors and is independent of the underlying molecular structure (Appendix *A* [App appa]). The evolution of the atomic electron configuration occupancies can be described by a system of coupled rate equations (Hau-Riege *et al.*, 2004 ▸) which account for various radiation damage processes: 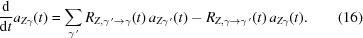
Here we have expanded the index α = {*Z*γ} to reflect the changes in occupancy of electronic configuration γ of element *Z*. The transition rate 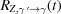 from configuration γ′ to configuration γ in general can be time-dependent. For biomolecules illuminated by XFEL pulses longer than 10 fs it has been demonstrated (Gorobtsov *et al.*, 2015 ▸) that photoelectrons and secondary electrons, produced by electron impact ionization and the Auger effect, make a substantial contribution to the dynamics of configuration occupancies *a*_*Z*γ_(*t*). We expanded the *AC4DC* atomic toolkit (Kozlov & Quiney, 2019 ▸; Kozlov *et al.*, 2020 ▸), which solves the atomic rate equations (16) with photoionization, Auger effect and fluorescence processes, to include electron impact ionization and three-body electron recombination. Here we modified the continuum model (Hau-Riege *et al.*, 2004 ▸; Gorobtsov *et al.*, 2015 ▸) to treat secondary electrons adiabatically as a Maxwellian plasma separately from the photoelectrons, which can collide with the former. The latter are described by two additional equations for their number and energy densities: 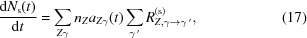


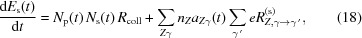


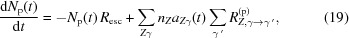


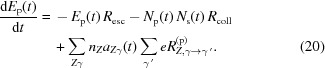
Here *N*_s_, *E*_s_ and *N*_p_, *E*_p_ are the number and energy density of the secondary and photoelectrons, respectively, *n*_*Z*_ is the number density of element *Z*, 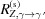 include those of the above-mentioned atomic processes that release or capture secondary electrons, 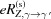 are the rates of the same processes weighted by their energy intake or release (see Appendix *B* [App appb] for details), and 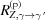 and 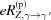 indicate the respective rates for photoelectrons. The temperature of secondary electrons is *T*(*t*) = 2*E*_s_(*t*)/3*N*_s_(*t*) (Boltzmann’s constant is equal to unity in atomic units). The photoelectron energy distribution is approximated by a delta function 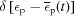, where the average photoelectron energy is 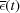 = *E*_p_(*t*)/*N*_p_(*t*). This leads to the loss of a major part of the high-energy tail of the total electron partition function (Hau-Riege, 2013 ▸), where electrons can acquire energies in excess of the incident photon energy ω. To capture this effect, a more sophisticated model (Leonov *et al.*, 2014 ▸) is required. In our model, collisions between photoelectrons are ignored, but their collisions with secondary electrons are included via the rate *R*_coll_ in the weak-beam approximation (Kunc, 1989 ▸) where the number density of secondary electrons exceeds that of photoelectrons by at least an order of magnitude. This model assumes that the finite thermalization time of photoelectrons is longer than the pulse length. This is a valid assumption for X-ray photon energies in excess of 2 keV and typical XFEL pulse parameters (Hau-Riege, 2013 ▸). We do not consider Coulomb trapping of photoelectrons at the later stages of the pulse, which is justified if the incident X-rays are sufficiently energetic and photoelectron scattering leaves them with a substantial portion of their initial kinetic energy (Gorobtsov *et al.*, 2015 ▸). Secondary electrons, which include electrons produced via Auger decay and impact ionization, are assumed to be trapped at all times during the pulse. This results in a spike in the secondary electron temperature at the early stages of the pulse, when only a few energetic secondary electrons are present. These electrons are assumed to undergo rapid thermalization and are trapped by the net positive charge of the sample that is the result of escaping photoelectrons, described by the rate *R*_esc_, which depends on the sample size (see Appendix *B* [App appb] for more details).

To estimate the accuracy of our atomic and plasma simulation model, *AC4DC*, we compare its predictions with the non-local thermodynamic equilibrium (*NLTE*) approach (Scott, 2001 ▸). The latter was initially developed for the description of laboratory plasmas and later adapted to model sample dynamics illuminated by an XFEL pulse (Caleman *et al.*, 2015*a* ▸; Jönsson *et al.*, 2015 ▸; Beyerlein *et al.*, 2018 ▸; Jönsson *et al.*, 2018 ▸). Two major differences between our approach and *NLTE* are the atomic structure description (*AC4DC* calculates electron transition rates for all possible ionic states) and the different dynamics of photoelectron thermalization (weak-beam approximation in *AC4DC*, instantaneous thermalization in *NLTE*). However, our model does not include continuum lowering that modifies the ionization potentials of atoms due to high-density electron plasma in the sample; this process is included in *NLTE*.

We compared average ionizations per atom of water and lysozyme under an XFEL pulse fluence ranging from 10^5^ to 10^6^ J cm^−2^ and a pulse length of 20 fs with an X-ray photon energy of 6 to 9 keV (Jönsson *et al.*, 2018 ▸). For the shortest pulse length available in the *NLTE* implementation (Jönsson *et al.*, 2018 ▸) the difference in prediction between the two models for the average charge per atom in lysozyme and water is under 10%. The secondary electron temperature predicted by *AC4DC* was higher for all but the weakest incident fluences compared with the temperature predicted by *NLTE*. The reason for this discrepancy is an amplification of Auger decay in ions, included in *AC4DC*, and continuum lowering, accounted for in *NLTE*. Finally, the accuracy of the rate equation model presented above depends on the relative scales of the photoelectron thermalization time and the XFEL pulse length. As the photoelectron thermalization time increases for higher X-ray photon energies, the approximation of the free-electron energy distribution that *AC4DC* relies on becomes more accurate.

## 4. Results   

At present, atomic resolution using XFEL sources remains a challenge. Imaging and reconstructing pre-designed DNA origami structures was proposed to guide the experimental and data analysis techniques required for the success of SPI (Xavier & Chandrasekaran, 2018 ▸). This approach was recently demonstrated in cryo-electron microscopy, where DNA origami was encapsulated into a capsid of simian vacuolating virus 40 (Kopatz *et al.*, 2019 ▸). These structures have a number of features that make them an attractive target for an SPI experiment, such as high reproducibility and homogeneity, structural robustness under various conditions, a chemical composition similar to that of most proteins, and a high degree of control over their structure (Xavier & Chandrasekaran, 2018 ▸; Kopatz *et al.*, 2019 ▸). While DNA origami structures may appear semi-periodic in small-angle scattering, they can be designed to resemble single molecules at wide angles. Here we apply the theoretical framework described above and implemented in *AC4DC* to investigate the coherence properties of scattering by DNA origami. First, we use information about the structure of the sample to solve the forward problem and simulate the first two coherent modes. Next, we use only the chemical composition (without the structural information) of the sample and the XFEL pulse parameters to construct the overlap matrix **S** and damage matrix **P**. This enables us to find the coefficients in equation (14) and obtain an approximation to the undamaged electron-density map. We consider incident X-ray photons at 8 keV in XFEL pulses of Gaussian shape with FWHMs ranging from 10 to 30 fs and fluences between 10^4^ and 10^7^ J cm^−2^. In our simulations we consider DNA origami that is spherical in shape with a radius of 20 nm, made up of equal proportions of adenine, guanine, cytosine and thymine nucleotides with a total number ratio of chemical elements of 39 (C):15 (N):24 (O):4 (P). The DNA strands occupy a volume equal to that of a cylinder with a radius of 1 nm and a length of 10 nm per 32 base pairs, with an additional 0.5 nm added to the radius to account for spacing between the strands (Rothemund, 2005 ▸).

Table 1 ▸ shows the fraction of photons in the first and second coherent modes given by equation (9) for the XFEL pulse parameters listed above.

**Figure 1:**
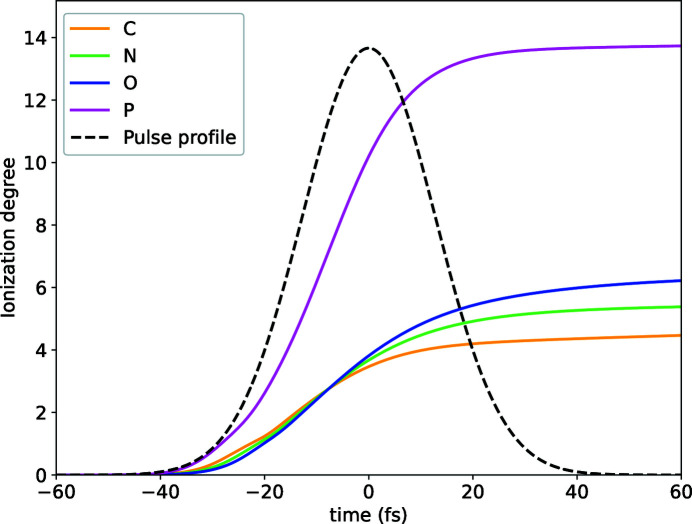
The average loss of bound electrons (ionization degree) for carbon, nitrogen, oxygen and phosphorus atoms in DNA origami, illuminated by a 30 fs XFEL pulse with a fluence of 3.2 × 10^7^ J cm^−2^.

We observed the largest decoherence for a 30 fs pulse with a fluence of 3.2 × 10^7^ J cm^−2^ (corresponding to a peak intensity of 1 × 10^21^ W cm^−2^). For these pulse parameters 91.7% of photons occupy the first mode and 8.0% occupy the second, with the remaining 0.3% occupying all other modes combined. Fig. 1 ▸ shows the ionization dynamics obtained by solving the system of equations (16) for atoms of the DNA origami exposed to a 30 fs XFEL pulse with a fluence of 3.2 × 10^7^ J cm^−2^.

**Figure 2:**
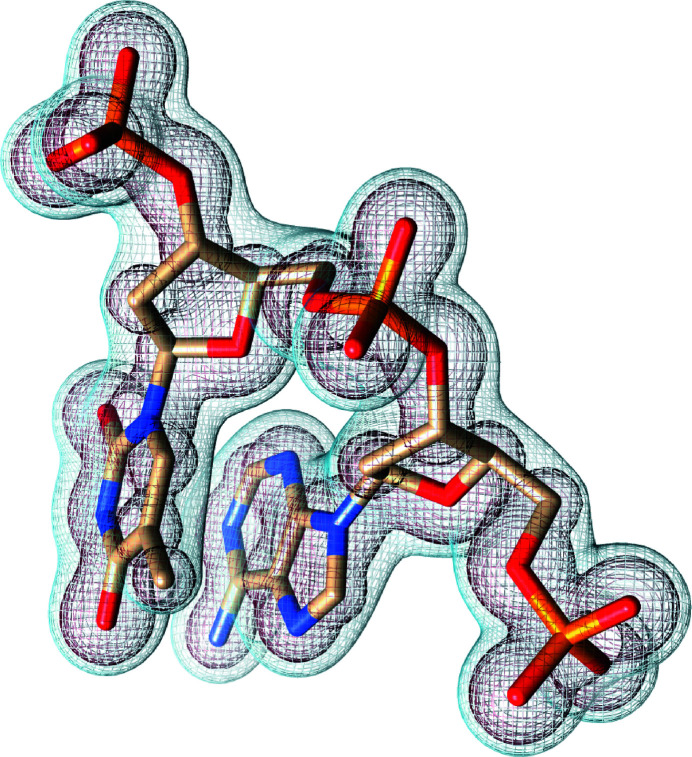
A simulation of the reconstructed electron density of a DNA fragment obtained from equation (14) using one (dark-red mesh) and two (pale-blue mesh) coherent modes. Values of constant electron density in both depicted surfaces are equal.

**Figure 3:**
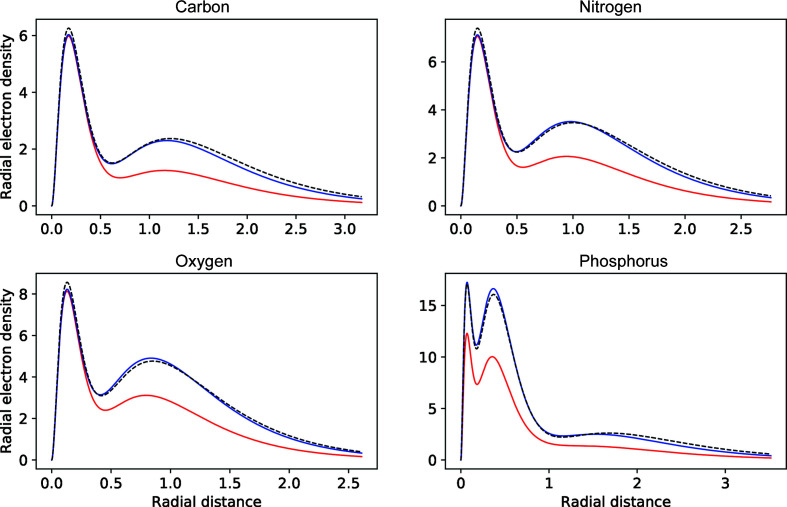
Radial atomic electron densities reconstructed using expansion (14) with two modes (blue lines) and one mode (red lines). Undamaged densities (black dashed lines) are shown for comparison. Radial distances from the atomic nucleus are expressed in Bohr radii, *a*_B_ = 0.529 Å, and the radial electron densities are in electrons per *a*_B_.

As is evident from Fig. 1 ▸, by the time the pulse reaches its peak almost half the electrons are stripped from their atoms due to ionization. The reduction in the number of bound electrons can be observed in Fig. 2 ▸ for an electron-density map (dark-red mesh) obtained from a single mode. In the diffraction data this reduction in scattering power is somewhat compensated by an increase in the number of incident photons for a higher incident fluence. The effective atomic radial densities, the Fourier transform of which gives pulse-averaged atomic form factors, are plotted in Fig. 3 ▸ in red for the dominant mode where they were scaled by a factor Ω^(*M*)^ [see equation (13) and the subsequent description]. The radial atomic electron density as depicted by the dominant coherent mode (which in the low-damage limit represents a pulse-averaged structure) has a relatively higher depletion of valence-shell electrons compared with the *K* shell, although the photoionization cross section is much higher for the latter. This occurs due to an enhancement of *K*-shell hole recombination in Auger decay in ions (Son *et al.*, 2011 ▸) and a stronger suppression of the contribution to the total wide-angle scattering from atoms that have lost a *K*-shell electron compared with atoms that have lost an electron from a valence shell.

## 5. Discussion and conclusions   

Incorporating modes beyond the dominant mode, which can be obtained from partially coherent diffraction images, enables us to obtain a reconstruction of the sample electron density (14) that is improved compared with the pulse-averaged density affected by radiation damage. This approach assumes that the motion of atoms in a sample during an XFEL pulse is small compared with their size, which may be achieved if the imaged molecule resides in a droplet illuminated with a short pulse (Hau-Riege *et al.*, 2007*b* ▸) but can be quite large otherwise (Fortmann-Grote *et al.*, 2017 ▸), especially for pulses well in excess of 30 fs (Martin *et al.*, 2015 ▸; Caleman *et al.*, 2015*b* ▸). The possibility of extracting coherent modes beyond the dominant one depends on the degree of coherence and the signal-to-noise ratio (SNR) in the average integrated intensity 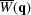. Indeed, since the number of photons in the second mode does not rise beyond 8% in our simulations, we expect at least an inversely proportional reduction in the SNR in the retrieved second mode compared with the first dominant mode. However, a sufficiently high number of diffraction patterns can provide the required SNR and will be achievable in the near future with the progress of XFEL technology (Sobolev *et al.*, 2020 ▸). Our results, shown in Table 1 ▸, indicate that for higher-fluence pulses rather substantial radiation damage is imprinted into the electron density of the sample. In such XFEL regimes the atomic form factors need to be adjusted using radiation damage models to prevent structure reconstruction algorithms from confusing neutral atoms with ions of different chemical elements. For higher pulse lengths and fluences we observe a relative reduction in the degree of coherence. For short pulses, higher coherence is observed due to the relative reduction in radiation damage associated with the contribution from secondary ionization processes (Hau-Riege *et al.*, 2004 ▸).

The impact of radiation damage on the reconstructed electronic structure of a fragment of DNA origami can be observed in Fig. 2 ▸. The electron density, depicted in dark red, was reconstructed using equation (14) from only the first coherent mode. The depletion of valence electrons observed in this figure is clearly visible when compared with the electron density reconstructed from both the first and second modes (light blue). Both reconstructions in Fig. 2 ▸ represent surfaces of constant electron density, the values of which are the same for both surfaces. The radial atomic electron densities plotted in Fig. 3 ▸ offer a deeper insight into the qualitative advantage of multi-mode electron-density reconstruction in the presence of radiation damage. The red and blue lines represent the 1D radial atomic electron densities used in the 3D electron-density surfaces of the corresponding colour in Fig. 2 ▸ within the framework of the independent-atom model. The two-mode reconstructions (blue) accurately reproduce the un­damaged radial electron densities, while the one-mode reconstructions (red) clearly show the depletion of valence electrons for all atoms except phosphorus, for which a more dramatic depletion is observed for all electrons including those in the *K* shell (first peak in radial density). Note that, in general, a single coherent mode reconstruction becomes dependent on the incident pulse parameters in the presence of significant radiation damage. This will introduce an additional complication into subsequent structure refinement. A two-mode reconstruction, on the other hand, is more robust to the choice of XFEL pulse parameters and yields a close approximation to the undamaged electron structure.

It is well known that elements as heavy as sulfur and phosphorus are ionized substantially faster by XFEL pulses than lighter elements of biological significance. The results in Fig. 1 ▸ indicate that phosphorus starts undergoing a rapid ionization several femtoseconds earlier than carbon, nitrogen and oxygen. Therefore, the radial electron density of phosphorus in Fig. 3 ▸, reconstructed using a single mode (red line), misses a larger fraction of initial electrons than lighter elements. Addition of the second mode into the reconstruction (blue line in Fig. 3 ▸) corrects phosphorus as well as the lighter elements. Therefore, the second coherent mode has an amplified contribution from heavier elements and a reduced contribution from lighter atoms compared with the first mode. In the context of single-particle imaging, this may have implications for imaging techniques that rely on anomalous diffraction from heavy elements, and this will be explored in future work.

## Figures and Tables

**Table 1:** Percentages of the total number of photons 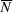 scattered by the sample that occupy coherent modes ψ_*k*_(**q**) Only values that correspond to the first (*k* = 0, top row for each pulse length) and second (*k* = 1, bottom row for each pulse length) coherent modes are depicted for a range of XFEL pulse lengths and fluences with an incident XFEL photon energy of 8 keV.

		Pulse fluence (× 10^4^ J cm^−2^)				
Pulse length (fs)	*k*	1	10	100	318	1000
10	0	99.98	99.46	96.52	94.66	94.65
	1	0.020	0.53	3.34	5.07	5.07
15	0	99.96	99.30	95.49	93.44	93.62
	1	0.032	0.69	4.33	6.25	6.00
20	0	99.96	99.15	94.69	92.63	93.02
	1	0.044	0.84	5.10	7.03	6.57
25	0	99.94	99.01	94.07	92.07	92.61
	1	0.055	0.96	5.70	7.57	6.96
30	0	99.93	98.89	93.56	91.65	92.31
	1	0.066	1.08	6.18	7.97	7.24

## Appendix A Coherent mode equations

In order to find the expression for coherent modes in (9) it is useful to consider the properties of the overlap matrix,

The product of the structure factor *T*_*Z*_(**q**) and atomic form factor *f*_*Z*γ_(*q*) is the Fourier transform of the sum of electron densities of atoms *Z* in a configuration γ,

Using Parseval’s theorem, the overlap matrix can be re-written as

The last part of this expression was obtained by neglecting atomic density overlap. An assumption of radial symmetry of the atomic electron density , followed by application of Parseval’s theorem, yields equation (15) for the overlap matrix. Any such matrix is Gramian (Horn & Johnson, 2013 ▸), and hence positive semi-definite, since  =  =  for any vector **x**. Moreover, the overlap matrix considered in the present paper is invertible, and therefore positive definite. Indeed, as we use a tight-binding model in which each atom of the sample is considered independent [equation (15)], the overlap matrix  is always equal to zero when *Z* ≠ *Z*′. This means that **S** has a block-diagonal structure, with each block corresponding to a different chemical element *Z*. Thus, its determinant can be zero only if the determinant of one of these individual blocks is zero. The latter condition would mean that the overlap matrix for different configurations of one atom is degenerate, which would correspond to a linear dependence of the atomic electron densities ρ_*Z*γ_(**r**). This is never the case for a set of atomic electron densities that correspond to different orbital occupancies (electron configurations), since the difference in the Coulomb interaction between electron configurations lifts degeneracy and therefore prevents linear dependence. Note that, in the atomic model (Kozlov & Quiney, 2019 ▸) used in this paper, we average over the total angular momentum and its projections, treating all these states in the ensemble {*Z*γ} as one state with an effective weight.

Let η_*k*_ and **g**_*k*_ be the eigenvalues and the orthonormal eigenvectors of the symmetric matrix **Q** = **S** ^1/2^ **PS** ^1/2^. Then for any vector **x** we have **Qx** = . As **P** = **S** ^−1/2^ **Q** **S** ^−1/2^, we obtain

Like the matrix **S**, the matrix **P** is also positive semi-definite, but unlike **S**, **P** can be highly degenerate. In particular, in the absence of radiation damage, the rank of **P** is equal to one, *i.e.* only one of the eigenvalues η_*k*_ is non-zero (in that case there is only one coherent mode, *i.e.* the diffracted wave is spatially fully coherent). Substituting equation (25) into the expression  in equation (6), where  is the vector with elements ρ_α_(**q**), we obtain the following representation of the mean diffracted intensity in terms of coherent modes and their mean ‘occupation numbers’ (Mandel & Wolf, 1995 ▸; Quiney, 2010 ▸; Quiney & Nugent, 2011 ▸; Lorenz *et al.*, 2012 ▸):

The functions ψ_*k*_(**q**) are orthonormal in the sense that

where we have used the orthonormality of vectors **g**_*k*_. It is also easy to verify that the vectors **v**_*k*_ = **S** ^−1/2^ **g**_*k*_ are eigenvectors of the matrix **PS**, *i.e.* **PSv**_*k*_ = η_*k*_ **v**_*k*_: the proof consists of substituting **g**_*k*_ = **S** ^1/2^ **v**_*k*_ into the eigenvector equation **S** ^1/2^ **PS** ^1/2^ **g**_*k*_ = η_*k*_ **g**_*k*_ and multiplying both sides by **S** ^−1/2^ from the left. Therefore, the coherent modes (9) are the scalar products of the electron-density vector and the eigenvectors of the matrix **PS**.

## Appendix B Atomic and electron plasma rate equations

This appendix describes the details of the rate-equation model for the description of atomic and electron plasma dynamics in an XFEL pulse. The rates in the electron plasma equations (17)–(20) are written as

where  is the Auger rate for element *Z*, which transitions from the initial electronic configuration γ to the final configuration γ′,  is the photoionization rate, ω is the photon energy and  is the ionization potential. The electron impact ionization (EII) and three-body recombination (TBR) rates are given by the following expressions:

where *f*(*p*) =  is the Maxwellian partition function for secondary electrons (normalized to unity),  =  = *E*_p_(*t*)/*N*_p_(*t*) is the average photoelectron speed, and  represents the rate of kinetic energy transfer from the photoelectron to the secondary electron it creates when ionizing an element *Z* in the initial configuration γ. The three-body recombination rate  was obtained using the principle of detailed balance (Landau & Lifshitz, 2002 ▸; Hau-Riege, 2011 ▸). For the electron impact ionization cross sections  and the differential cross section  we used the binary-encounter Bethe model (Kim & Rudd, 1994 ▸).

The escape rate of photoelectrons from the sample is accounted for by the rate *R*_esc_ =  in equations (19) and (20), where *r* is the radius of a spherical sample and  = . In the derivation of *R*_esc_ it was assumed that the photoelectrons are distributed uniformly in the sample and have a random, but uniformly distributed, direction of their speed .

Prior to numerical solution of the atomic (16) and plasma rate equations (17)–(20), all atomic configurations γ are calculated for all atoms *Z*. The atomic rates and the respective blocks of the overlap matrix are calculated and stored prior to solving the rate equations. During solution of the rate equations, at every time step the integrals in (33) and (36) are calculated using 13-point Gaussian quadrature. The two remaining rates, secondary electron recombination (34) and photoelectron impact ionization (35), are calculated at each time step as well, using the atomic parameter stored at the previous step.
